# Descriptive Characteristics and Health Outcomes of the Food by Prescription Nutrition Supplementation Program for Adults Living with HIV in Nyanza Province, Kenya

**DOI:** 10.1371/journal.pone.0091403

**Published:** 2014-03-19

**Authors:** Jason M. Nagata, Craig R. Cohen, Sera L. Young, Catherine Wamuyu, Mary N. Armes, Benard O. Otieno, Hannah H. Leslie, Madhavi Dandu, Christopher C. Stewart, Elizabeth A. Bukusi, Sheri D. Weiser

**Affiliations:** 1 School of Medicine, University of California, San Francisco, San Francisco, California, United States of America; 2 Department of Obstetrics, Gynecology and Reproductive Sciences, University of California, San Francisco, San Francisco, California, United States of America; 3 Division of Nutritional Sciences, Cornell University, Ithaca, New York, United States of America; 4 Family AIDS Care and Education Services, Center for Microbiology Research, Kenya Medical Research Institute, Nairobi, Kenya; 5 Division of Epidemiology, University of California, Berkeley, Berkeley, California, United States of America; 6 Division of Hospital Medicine, University of California, San Francisco, San Francisco, California, United States of America; 7 Department of Pediatrics, University of California, San Francisco, San Francisco, California, United States of America; 8 Division of HIV/AIDS, University of California, San Francisco, San Francisco, California, United States of America; Asociacion Civil Impacta Salud y Educacion, Peru

## Abstract

**Background:**

The clinical effects and potential benefits of nutrition supplementation interventions for persons living with HIV remain largely unreported, despite awareness of the multifaceted relationship between HIV infection and nutrition. We therefore examined descriptive characteristics and nutritional outcomes of the Food by Prescription (FBP) nutrition supplementation program in Nyanza Province, Kenya.

**Methods:**

Demographic, health, and anthropometric data were gathered from a retrospective cohort of 1,017 non-pregnant adult patients who enrolled into the FBP program at a Family AIDS Care and Education Services (FACES) site in Nyanza Province between July 2009 and July 2011. Our primary outcome was FBP treatment success defined as attainment of BMI>20, and we used Cox proportional hazards to assess socio-demographic and clinical correlates of FBP treatment success.

**Results:**

Mean body mass index was 16.4 upon enrollment into the FBP program. On average, FBP clients gained 2.01 kg in weight and 0.73 kg/m^2^ in BMI over follow-up (mean 100 days), with the greatest gains among the most severely undernourished (BMI <16) clients (p<0.001). Only 13.1% of clients attained a BMI>20, though 44.5% achieved a BMI increase ≥0.5. Greater BMI at baseline, younger age, male gender, and not requiring highly active antiretroviral therapy (HAART) were associated with a higher rate of attainment of BMI>20.

**Conclusion:**

This study reports significant gains in weight and BMI among patients enrolled in the FBP program, though only a minority of patients achieved stated programmatic goals of BMI>20. Future research should include well-designed prospective studies that examine retention, exit reasons, mortality outcomes, and long-term sustainability of nutrition supplementation programs for persons living with HIV.

## Introduction

The human immunodeficiency virus (HIV), undernutrition, and food insecurity remain leading causes of morbidity and mortality in sub-Saharan Africa despite increases in international funding for care and improved access to highly active antiretroviral therapy (HAART) [1], [2]. Undernutrition and food insecurity converge to exacerbate the detrimental health consequences of HIV through complex feedback cycles [1], [3] and disproportionately affect the estimated 22.5 million persons living with HIV/AIDS in sub-Saharan Africa [4]. HIV infection amplifies undernutrition by weakening metabolic function through impaired storage and utilization of nutrients, reduced absorption of carbohydrates and fats (including fat-soluble vitamins), and recurrent diarrhea from compromised immunity [1], [5]. In addition, persons living with HIV have increased protein and energy requirements due to increased resting energy expenditure as a function of HIV load [6], [7]. Weight loss [8] and undernutrition [9], [10] are well-established predictors of mortality in HIV-infected patients, independent of CD4 lymphocyte counts.

Various nutritional interventions have been developed for persons living with HIV, including food-based supplement interventions, oral supplements with specific micronutrients, enteral or total parenteral therapy, and appetite stimulants [11]. In sub-Saharan Africa, food-based nutrition supplementation is regularly used to complement locally available foods to provide adequate daily macronutrient and micronutrient intake for persons living with HIV [2]. One study found that 90% of HIV programs across nine African countries provided some form of nutrition support [12]. Common supplements include corn-soy blends, fortified blended foods, and high-energy ready-to-use therapeutic foods [2].

In spite of knowledge of the multifaceted relationship between HIV infection and nutrition [1] as well as funding for supplementation programs [13], the quantitative clinical effects and potential benefits of macronutrient supplementation interventions to persons living with HIV remain largely unknown [14], [15]. Indeed, a 2007 Cochrane systematic review on the effect of macronutrient supplementation on morbidity and mortality in persons living with HIV found no relevant clinical trials that reported on mortality, morbidity, or HIV disease progression [11]. The review did identify studies evaluating BMI change and found no significant effects of macronutrient supplementation on BMI or weight gain [11], [16]. However, one recent trial in Malawi demonstrated that patients receiving ready-to-use fortified spread had significantly greater increase in BMI than patients receiving corn-soy blend over a 14-week intervention period, though there was no control study arm without nutritional supplementation [17]. One Haitian study found that food assistance was associated with significantly improved BMI and adherence to clinic visits at 12 months [18]. Limited data exist to guide targeted food programs to identify individuals with the greatest potential to benefit, appropriate enrollment criteria, optimal duration, and appropriate exit criteria for nutrition supplementation programs for persons living with HIV/AIDS. Multiple review articles have highlighted the critical importance of additional research in these areas [1], [2], [11].

In response to these calls, we examined the Food by Prescription (FBP) program, a nutrition supplementation program for persons living with HIV/AIDS in Nyanza Province, Kenya. The objectives of this study were to: 1) describe the demographic and health characteristics of patients enrolled in the FBP program, 2) describe programmatic outcomes including duration of enrollment in FBP, weight change, BMI change, and attainment of BMI≥20; and 3) identify demographic and health factors associated with attainment of BMI≥20.

## Materials and Methods

### Study setting and program description

Nyanza Province has the highest HIV prevalence in Kenya, at 15.1% [19]. Nearly universal food insecurity among persons living with HIV/AIDS has been reported in rural Nyanza Province [20], [21]. Furthermore, 34% of men and 25% of women living with HIV in Nyanza Province are underweight (BMI<18.5) [22].

FBP was established in 2006 as a public-private partnership among the National AIDS and STI Control Programme in the Kenyan Ministry of Health, the United States Agency for International Development, and Insta Products Ltd., a Kenyan food manufacturing company. FBP operates in all regions of Kenya and reached 27,913 clients by 2007 [23]. The program includes nutritional screening, assessment, education, counseling, and provision of fortified corn-soy flour supplements to malnourished adults living with HIV, HIV-positive pregnant and postpartum women, and malnourished orphans and vulnerable children. The FBP adult supplement, called *Foundation Plus*, consists of nutrient-dense, pre-cooked whole maize, soybeans, vegetable oil, and cane sugar, fortified with vitamins and minerals in varying quantities that is rehydrated in boiling water and consumed as porridge by participants. This provides the majority of patients' daily micronutrient requirements and approximately one-half of clients' average daily energy requirement (450 kcal total and 15 g of protein per 100 g of *Foundation Plus*).

Adult (defined as age 15 or older) enrollment criteria for this FBP program study included having a body mass index (BMI) <18.5 kg/m^2^ among patients enrolling into HIV care. The goal of the program is to restore patients to healthy nutritional status; FBP program guidelines state that adults should be discharged when their BMI improves to ≥ 20 kg/m^2^ [23]. The program discharge criteria of BMI>20 was designed to help clients accrue buffer weight above BMI of 18.5 to prevent relapse in the FBP program [23].

For this retrospective cohort study, we obtained electronic FBP program encounter records for all non-pregnant adult patients who enrolled into the FBP program with BMI <18.5 kg/m^2^ at all 48 Family AIDS Care and Education Services (FACES) sites in Nyanza Province between 1 July 2009 and 31 July 2011. We limited inclusion to those with a minimum of one follow-up visit.

### Data collection

This study was conducted at FBP distribution sites supported by FACES, a collaboration between the Kenya Medical Research Institute (KEMRI) and the University of California, San Francisco (UCSF) that provides HIV prevention, care, and treatment services in Nyanza Province. At all FACES-supported sites offering FBP, trained community and clinic health assistants collected demographic information including gender, age, marital status, and household size using a standardized intake form upon enrollment. Patients' weight and height, World Health Organization (WHO) adult staging category [24], CD4 T-cell count, and HAART status were also determined at enrollment and at follow-up visits, generally scheduled one month apart. Patients were classified as severely underweight (< 16), moderately underweight (≥16.5 to <17), mildly underweight (≥17 to <18.5), normal weight (≥18.5 to <25), or overweight (BMI ≥ 25) using criteria from the Food and Agriculture Organization of the United Nations [25].

### Ethics statement

The study was approved by the Ethical Review Committee at the Kenya Medical Research Institute and the Committee on Human Research at the University of California, San Francisco. The data were analyzed anonymously. A waiver of informed consent for adults and minors was specifically granted by the approving institutional review boards.

### Data management and statistical analysis

Patient FBP encounter records were entered and stored in Microsoft Access 2007, subsequent data management was performed using STATA 12 (StataCorp LP, College Station, TX), and analysis was conducted using SPSS 12.0 (SPSS Inc., Chicago, IL). Any individual with at least two visits within a six month timeframe was included in analyses. If there was a more than six month gap between two visits, the latter visit and all subsequent visits were considered part of a new entry and all data (baseline values, duration, BMI change) were calculated separately for each entry. Duration of FBP was defined as days from FBP enrollment to attainment of BMI >20 or time of censoring. Individuals not reaching BMI >20 were censored at date of exit or last recorded appointment.

Differences in selected programmatic outcomes of FBP participants (including duration of FBP, weight change, BMI change, and attainment of BMI>20 as well as other BMI values) were analyzed by baseline BMI using ANOVA and Pearson's chi square tests, and by HAART status using independent samples t-tests. Cox proportional hazards regression was used to build a model in which the outcome was time until event, in this case, time in the FBP program until a participant achieved a BMI >20. For this evaluation our goal was to determine how long a patient spent in the program before achieving the desired outcome, as well as the clinical and demographic predictors of the outcome. Survival curves were used to evaluate factors associated with time to FBP treatment success. The associations between predictors and time to treatment success were analyzed using bivariate and multivariate Cox proportional hazards regression. Predictor variables were measured at baseline and included age (continuous), gender (male versus female), household size (continuous), marital status (categorical: single, married, divorced, widowed), BMI classification (categorical: severely, moderately, or mildly underweight, normal weight), antiretroviral therapy status (receiving or not receiving HAART during the study period, eligibility based on immunologic criteria of CD4 <350 to receive HAART), CD4 count (continuous), and WHO Stage (categorical: stages 1, 2, 3, and 4) [24]. All predictor variables were included in adjusted models. Hazard ratios (HR) and 95% confidence intervals (CI) were calculated from the Cox proportional hazards regression model. All variables were checked for multicollinearity and had a variance inflation factor <2. Models were tested for violations of the proportional hazards assumption and time varying covariates were used when the proportional hazards assumption was not met. All p values were two-tailed, with the level of significance set at p<0.05.

## Results

Overall, 1,017 individuals met the study eligibility criteria. Mean age was 36, a majority of the sample were female, approximately half were married, and mean household size was four (Table 1). The average BMI was 16.4 at the time of entry into the FBP program. Mean CD4 count was 259 at baseline and a majority of the sample received HAART during the study period.

**Table 1:** Selected demographic and health characteristics of study participants at enrollment to Food by Prescription in Nyanza Province, Kenya.

	n	Mean or %	SD	Range
*Demographic characteristics*				
Age (years)	1017	36.4	11.4	16–81
Gender	998			
Male	355	34.8%		
Female	643	63.1%		
Household size	869	4.4	2.3	0–42
Marital status	990			
Single	146	14.3%		
Married	528	51.8%		
Divorced	96	9.4%		
Widowed	220	21.6%		
*Health characteristics*				
BMI at baseline (kg/m^2^)	987	16.4	1.5	11.0–18.5
BMI classification	987			
Severely underweight (BMI <16)	361	35.4%		
Moderately underweight (16 ≤ BMI <17)	244	23.9%		
Mildly underweight (17 ≤ BMI <18.5)	382	37.5%		
CD4 at baseline (cells/mm^3^)	712	258.8	237.5	1–1848
WHO stage at baseline	932			
Stage 1	86	8.4%		
Stage 2	242	23.7%		
Stage 3	459	45.0%		
Stage 4	145	14.2%		
Highly active antiretroviral therapy (HAART) status	1015			
Pre-HAART	339	33.3%		
Received HAART during study period	676	66.3%		

**Figure 1:**
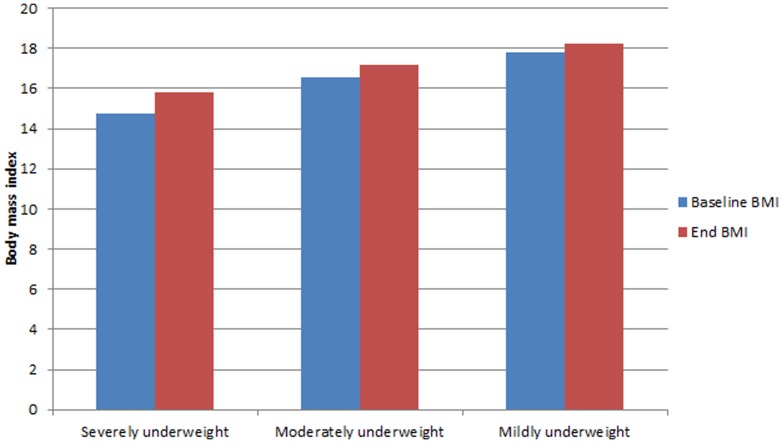
BMI at baseline and end of FBP, stratified by baseline BMI classification.

Mean and median duration of FBP were 100 and 62 days, respectively. Differences in FBP duration varied by baseline BMI (Table 2). The mean weight gain was 2.01 kg during FBP treatment. Patients classified as severely underweight gained significantly more weight than moderately underweight and mildly underweight patients (Figure 1). Overall, 13.1% of participants reached a BMI of 20, the threshold for discharge from the FPB program, while nearly half of participants achieved a BMI increase ≥0.5. Overall, FBP participants experienced an increase in CD4 count of 54 cells/mm^2^. Participants enrolling with lower BMI had lower baseline CD4 counts; however, there was no statistically significant difference in CD4 count change by BMI category over the study period.

**Table 2:** Programmatic outcomes of Food by Prescription nutrition intervention by BMI classification.

			BMI classification at baseline				
			Severely underweight	Moderately underweight	Mildly underweight		
	Overall	Range	BMI <16	BMI ≥16.5 to <17	BMI ≥17 to <18.5	Test statistic	p value
Number of subjects	965		361	244	382		
Outcome, mean (SD)							
Duration of FBP (days)	99.90 (112.90)	30 – 583	103.43 (119.05)	105.36 (122.40)	94.27 (100.95)	0.92^a^	0.399
Weight change during FBP (kg)	2.01 (4.67)	−18.00 – 30.00	3.06 (5.30)	1.64 (4.89)	1.26 (3.62)	14.99^a^	<0.001
BMI change during FBP (kg/m^2^)	0.73 (1.69)	− 5.56 – 11.02	1.09 (1.90)	0.61 (1.76)	0.46 (1.33)	13.76^a^	<0.001
Achieved BMI>20, n (%)	129 (13.1%)		30 (8.3%)	34 (13.9%)	65 (17.0%)	12.59^b^	0.002
Achieved BMI≥18.5, n (%)	215 (22.2%)		38 (10.8%)	45 (19.1%)	132 (34.9%)	63.52^b^	<0.001
Achieved BMI increase ≥5%, n (%)	359 (37.1%)		162 (45.9%)	87 (36.9%)	110 (29.1%)	22.06^b^	<0.001
Achieved BMI increase ≥0.5, n (%)	430 (44.5%)		180 (51.0%)	101 (42.8%)	149 (39.5%)	10.08^b^	0.006
CD4 count, mean (SD)							
CD4 count at baseline (cells/mm^3^)	258.83 (237.48)	1 – 1848	226.02 (230.06)	242.72 (229.28)	295.54 (244.80)	6.13^a^	0.002
CD4 change during FBP (cells/mm^3^)	54.33 (217.52)	−1440 – 1041	28.28 (243.65)	81.53 (211.40)	63.30 (204.07)	0.662^a^	0.518

a F test from ANOVA

b Pearson's chi square test

**Figure 2:**
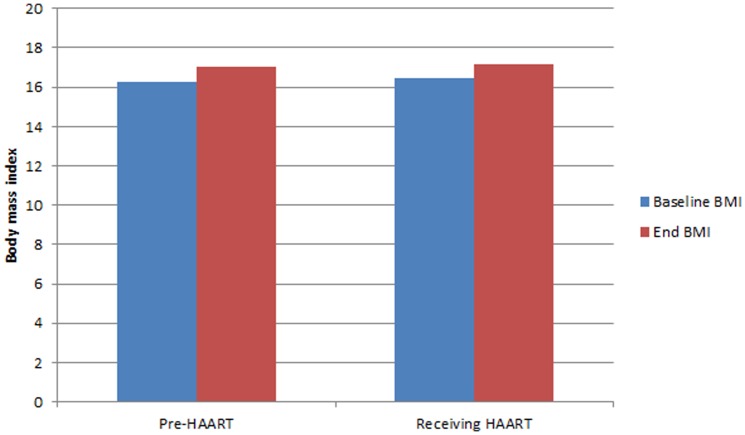
BMI at baseline and end of FBP, stratified by HAART status.

Patients not requiring HAART per CD4 guidelines as well as those receiving HAART both gained weight with no significant differences based on HAART status (*t* = −0.848, p = 0.40, Figure 2). Patients not attaining BMI >20 exited without a recorded reason (60.3%), were still active in treatment (14.4%), deceased (3.0%), considered treatment failure (1.7%), or referred to another facility (0.8%).

In the multivariate model, younger age, male sex, greater BMI, and not requiring HAART during the study period were strongly associated with an increased rate of attaining a BMI>20 (Table 3). For each additional year of age, patients had a decreased rate of attaining a BMI>20 while receiving FBP. Compared with men, women had a decreased rate of attaining BMI>20, controlling for all other predictor variables.

**Table 3:** Factors associated with time to attainment of BMI>20 for patients receiving Food by Prescription.

		Bivariate analysis		Multivariate analysis	
Variable	n (%)	HR (95% CI)	p value	AHR (95% CI)	p value
Age	1017	0.98 (0.96–0.99)	0.008	0.97 (0.94–1.00)	0.029
Gender	998				
Male	355 (34.8%)	1 [Reference]		1 [Reference]	
Female	643 (63.1%)	0.57 (0.40–0.81)	0.002	0.51 (0.30–0.86)	0.012
Household size	869	0.97 (0.88–1.07)	0.568	1.00 (0.87–1.14)	0.954
Marital status	990		0.970		0.480
Single	146 (14.3%)	0.98 (0.55–1.73)	0.932	0.82 (0.36–1.86)	0.63
Married	528 (51.8%)	0.92 (0.59–1.43)	0.71	0.61 (0.31–1.21)	0.158
Divorced	96 (9.7%)	1.03 (0.53–2.04)	0.914	0.87 (0.31–2.43)	0.796
Widowed	220 (21.6%)	1 [Reference]		1 [Reference]	
Baseline BMI	987	1.29 (1.12–1.48)	<0.001	1.52 (1.23–1.86)	<0.001
CD4^a^	712	1.00 (1.00–1.00)	0.202	1.00 (1.00–1.00)	0.093
WHO stage	932		0.51		0.075
Stage 1	86 (8.4%)	0.82 (0.41–1.63)	0.566	0.63 (0.23–1.69)	0.356
Stage 2	242 (23.7%)	0.67 (0.39–1.15)	0.142	0.34 (0.15–0.80)	0.013
Stage 3	459 (45.0%)	0.75 (0.47–1.21)	0.235	0.76 (0.39–1.49)	0.422
Stage 4	145 (14.2%)	1 [Reference]		1 [Reference]	
HAART status	1015				
Pre-HAART	339 (33.3%)	2.54 (1.77–3.64)	<0.001	4.18 (2.42–7.22)	<0.001
Receiving HAART	676 (66.3%)	1 [Reference]		1 [Reference]	

Abbreviations: HR, Hazard ratio; AHR, Adjusted hazard ratio; CI, Confidence interval; HAART, Highly active antiretroviral therapy

a Time-varying covariate used in multivariate model.

**Figure 3:**
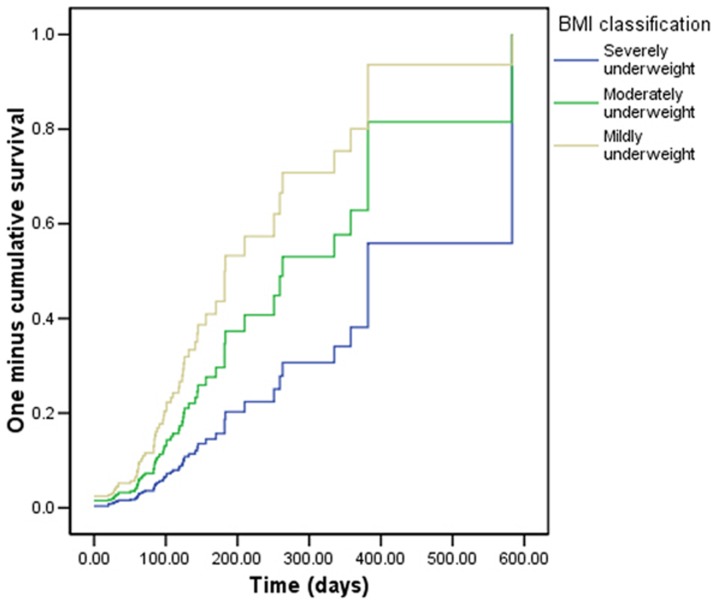
Survival curve of time to BMI > 20, by BMI classification.

**Figure 4:**
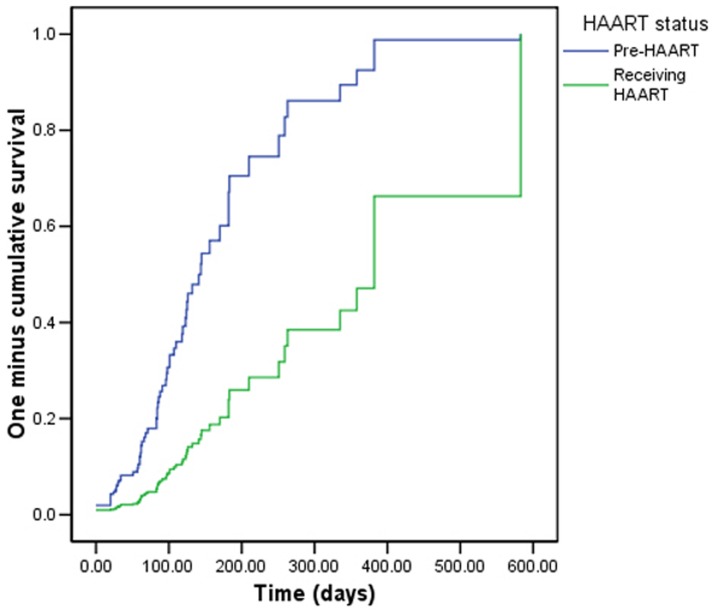
Survival curve of time to BMI > 20, by HAART status.

Higher baseline BMI was associated with a higher rate of attaining BMI>20 (Table 3, Figure 3). Compared to patients receiving HAART, patients who did not qualify for and therefore did not receive HAART during the study period had an increased rate of attaining BMI>20 (Table 3, Figure 4). Household size, marital status, CD4 count, and WHO stage were not significantly associated with time to attainment of BMI>20 in bivariate and multivariate Cox proportional hazards regression models.

## Discussion

Patients receiving FBP had significant increases in weight and BMI, with the greatest gains among the most severely undernourished clients. Yet only 13.1% of clients attained a BMI >20, the program criteria for discharge. Higher nutritional status at baseline, younger age, male sex, and not requiring HAART during the study period were associated with a higher rate of attainment of BMI>20.

FBP program participants gained an average of 2 kg during the duration of their FBP treatment and increased their BMI by 0.73 kg/m^2^. This is consistent with the findings of weight and BMI gains from a corn-soy supplementation trial in Malawi [17] and a food assistance program in Haiti [18], but stands in contrast to studies in Europe [26] and the USA [27], [28] that did not find significant effects of macronutrient supplementation on BMI or weight gain [11], [16].

The most severely underweight clients had the greatest gains in weight and BMI, given that they had the most opportunity for improvement. This may argue for prioritization of food supplements to participants with the lowest BMIs if food supplementation is limited. However, because participants in the severely underweight category also started with the lowest BMI at baseline, they never attained an absolute BMI as high as those who started in higher BMI categories. Therefore, those who started with the highest BMI had the best endpoint weights. Severely malnourished clients also received the longest duration of therapy compared to other less undernourished groups. This suggests that FBP programs might consider a longer duration or higher calorie content of nutritional supplementation for severely malnourished clients.

Overall, 13% of clients attained the FBP program discharge criteria of BMI>20. The program discharge criteria of BMI>20 was designed to help clients accrue buffer weight above BMI of 18.5 to prevent relapse in the FBP program [23]. Therefore, a larger percentage (22.2% attaining BMI>18.5) of clients attained a “normal weight” status than were actually discharged from the program (13.1% attaining BMI>20). These low rates of attainment may indicate that the program's set discharge criteria are unrealistic to attain for most participants. However, nearly half of participants achieved a BMI increase of ≥0.5, which may be a more attainable target outcome. Alternative BMI outcome goals may be considered in the future.

There are several potential reasons for low rates of attainment of program discharge criteria. Patients living in households with less food availability may be more likely to share their FBP with children and other members of their household, reducing the effect of supplementation on their own BMI. Furthermore, the FBP intervention was designed to supplement an existing diet, rather than to provide a primary food source [13], but participants living with severe food insecurity may not have regular access to other adequate food. Longer duration or higher caloric content of supplementation may also be considered. In addition, many patients exited before achieving target outcomes. Some potential reasons for exiting or lost to follow-up reported in other FBP studies include health workers and participants being unclear of the protocol (clients discharged after gaining some weight or after certain duration of time), lack of documentation in the medical record, patient dislike of the product, and the burden of transportation costs [13]. Additional exit reasons identified in the present study included lost to follow-up, nutritional deterioration, food allergies, transfer of care to another facility, or death. Participants receiving HAART had lower rates of lost to follow-up than participants not receiving HAART, even while receiving food supplementation. Furthermore, high rates of lost to follow-up have been reported in other FBP programs, at 50.1% in Kenya [23] and 70.6% in Ethiopia [13]. Future research should further explore clients' reasons for exiting the FBP program prior to attainment of program goals to guide future policies and ensure program objectives are consistent with what is being practiced in the field.

Clients not receiving HAART during the study period had a higher rate of attainment of BMI >20 compared with clients receiving HAART. This may be because individuals on HAART had more advanced disease at study enrollment (CD4<350 was eligibility criteria for receiving HAART) or alternatively that individuals on HAART had poorer adherence to FBP due to gastrointestinal side effects as reported elsewhere [23]. Though many nutrition support programs often specifically target individuals receiving HAART [1], our data suggest that nutrition supplementation can be beneficial for persons not yet requiring HAART by potentially increasing CD4 count.

Younger age was significantly associated with a greater rate of achievement of BMI>20. Younger persons may be more resilient and responsive to nutritional supplementation than the elderly. Several studies have reported higher food insecurity levels among the elderly due to challenges in completing agricultural labor, difficulty accessing alternate sources of food, dependence on others for food, and having additional medical co-morbidities [20], [29]–[31]. The elderly who are physically weak or disabled may also have a greater challenge carrying and transporting large amounts of heavy food supplements [23]. They may additionally have lower success rates due to compromised immunity and reluctance to follow the FBP instructions. Though FBP is intended only for the individual client, there may be differences in how persons at different stages of life share and allocate their food within their household based on level of perceived social responsibility for others, seniority in the household, capacity to perform labor and generate income, or other factors [1], [23].

Men had a higher rate of achieving BMI>20 than women. Men on average enroll later into HIV care with more advanced HIV progression and lower BMI than women [22], which one would expect to predict poorer nutritional outcomes. However, women may be more likely to be non-adherent to FBP due to sharing of food at home with other family members, particularly to young dependents or HIV-positive spouses, due to their central role in household food resource allocation and perceived social obligation [23], [32]. Sharing of food supplements at home may be the most important contributor to non-adherence with FBP and the reason for inadequate nutrient intake [23] particularly among women. The issue of food sharing should be carefully explored in future studies and addressed in nutrition guidelines. In particular, future household-level research could investigate the impact of FBP on individuals living in households in which single versus multiple members were receiving FBP.

Though this study only included FBP participants who enrolled based on strict BMI criteria (BMI <18.5), participants may also qualify with a mid-upper arm circumference (MUAC) <22 cm if BMI is not available. Clinics in resource-limited settings may not have access to well calibrated scales and height measuring boards. MUAC is an alternate measurement used by the FBP program for enrollment in the absence of BMI data and may be a viable alternative to BMI as it does not require expensive equipment or mathematical calculations for assessment of nutritional status [33]. Future research should examine nutritional and health outcomes of participants who enroll using MUAC criteria in comparison to those who enroll with BMI criteria. Furthermore, in practice, patients with a BMI ≥18.5 deemed to be clinically malnourished are occasionally enrolled for FBP nutrition support, a phenomenon that should additionally be explored in future investigations.

This study has several limitations. Not all patient charts were complete, which led to missing data. Selection bias may have occurred as we only included patients with longitudinal data (minimum of one follow-up visit); however, there were no significant differences in demographic or health characteristics between patients who only had one recorded visit and those with more than one visit (data not shown). Patients were followed through their last recorded appointment; however, many patients did not have a recorded exit reason and therefore were lost to follow-up before attaining program discharge criteria of BMI >20. Though participants' demographics ranged widely in terms of age and household size, these variables were overall normally distributed and likely represented true variation possibly seen in other parts of sub-Saharan Africa. Due to regular shortages in FBP supply, it is possible that some patients did not receive FBP for their entire recorded duration of treatment. Although useful for international comparison, BMI has limitations in measuring undernutrition, body fat, and visceral adipose tissue, particularly among non-European populations [34], [35]. Finally, there was no comparison group of persons who did not receive FBP nutrition supplementation, and thus our longitudinal findings remain descriptive.

Despite these limitations, the relatively large sample size, the demographic similarities of FBP clientele to the overall HIV patient population in Nyanza Province [22], and the implementation of the FBP program throughout Kenya and other parts of sub-Saharan Africa in regions with similar documented rates of undernutrition [22], [36], [37] supports the generalizability of this study's findings.

In sum, athough we found weight and BMI gain among FBP participants, only 13% of participants met stated program goals. Thus, there is critical need for well-designed and adequately-powered studies evaluating the effect of nutrition supplementation on clinical outcomes and mortality for adults living with HIV in order to guide global policy. Future research should examine the longer-term effects and sustainability of nutrition supplementation programs. Furthermore, understanding the complementary role of agricultural, educational, and poverty reduction interventions may be imperative to the success and longevity of sustainable nutrition programs for persons living with HIV.
